# Multi-Mode Face-to-Face and Telephone Approach to Data Collection in Health Surveys: A Scoping Review

**DOI:** 10.3390/epidemiologia5040054

**Published:** 2024-12-19

**Authors:** Paulo Henrique Guerra, Letícia Aparecida Calderão Sposito, Daniel Umpierre, Alex Antonio Florindo

**Affiliations:** 1 Institute of Biosciences, São Paulo State University (UNESP), Rio Claro 15385-000, Brazil; 2 Department of Physical Education, Federal University of Santa Catarina, Florianopolis 88040-900, Brazil; sposito.ef@gmail.com; 3 Postgraduate Program of Health Sciences, Federal University of Rio Grande do Sul, Porto Alegre 90035-007, Brazil; daniel.umpierre@gmail.com; 4 LADD Lab, Hospital de Clínicas de Porto Alegre, Centro de Pesquisa Clínica, Porto Alegre 90035-007, Brazil; 5 School of Arts, Sciences and Humanities, University of São Paulo, São Paulo 05508-070, Brazil; aflorind@usp.br; 6 Physical Activity Epidemiology Group, University of São Paulo, São Paulo 05508-070, Brazil

**Keywords:** data collection, interviews as topic, telephone, health surveys, review

## Abstract

**Background/Objectives**: The present study aims to identify information from health research studies that have used a mixed-methods data collection approach, considering the combination of face-to-face and telephone interviews (referred to as the MMFT approach), specifically focusing on identifying themes, objectives, designs, populations involved, and implementation processes. **Methods**: A scoping review was developed, with systematic searches performed in March 2024 across five databases, namely, PubMed; SciELO; Scopus; Web of Science; as well as Google Scholar and reference lists. The inclusion criteria were defined under the following categories: “Participants” (observational epidemiological studies, with no restrictions as to where they were carried out, sampling techniques, or sample profiles); “Concept” (use of MMFT in data collection), and “Context” (studies carried out in the health area, with no restrictions on the theme/subject). The review process was carried out by three researchers who worked independently. **Results**: From the initial 1515 potential references, the synthesis of this review included data from seven original studies, highlighting cross-sectional designs, involvement of adults and/or elderly people without specific health conditions, variability between data collection strategies, and complementary use of online approaches. **Conclusions**: Based on the evidence generated, it is recommended that future studies assess aspects of the MMFT approach in terms of response rate, cost reduction, and increasing the speed of conducting health surveys.

## 1. Introduction

Data collection is an essential process in scientific studies as it involves generating the data needed for validation. The biases related to this process include “non-response” [1] and “non-coverage” [2], which, respectively, refer to the non-participation of people selected in the sampling process (e.g., due to refusal, or even some type of inability to participate in the study) and the exclusion of certain audiences in the sample, generally due to the lack of access or opportunity to participate in the study.

Previous surveys suggest that non-respondents or those with incomplete data have poorer socioeconomic and health conditions compared to respondents [1,3]. Furthermore, excluding people without a telephone line in face-to-face surveys can distort the results as more vulnerable profiles would be omitted [4].

Recognizing that data obtained from health surveys can vary depending on the data collection method [5], there is a need to adopt strategies that facilitate the participation of all people selected in the sampling process. One emerging strategy involves the use of mixed-mode data collection, which combines various approaches, such as face-to-face, telephone calls, or smartphones, in order to expand the opportunities for the participation of people selected in the sampling process [6,7,8]. Even with the different strategies employed in the multi-mode approach to data collection [6,7], we highlight the potential of approaches that incorporate both face-to-face and telephone interviews (referred to as MMFT) [8].

Even though the ways in which telephones are used have expanded since the advent of mobile telephony and the commercialization of smartphones, the complementarity of the MMFT approach can, as mentioned above, facilitate a research team’s access to people, reduce costs, and make it possible to obtain a more realistic (or less biased) estimate of the situation of interest. These premises support and justify conducting a study that can summarize the information to aid future projects involving MMFT data collection.

In order to identify the characteristics and strategies, as well as strengthen the decision-making for an original project, the present study aims to identify information from health research studies that have used MMFT, specifically focusing on identifying the themes, objectives, designs, populations involved, and implementation processes.

## 2. Materials and Methods

### 2.1. Design

This study is part of a larger study, entitled “Health Survey of Sao Paulo: Physical Activity and Environment” (“Inquérito de Saúde de São Paulo-ISA: Atividade Física e Ambiente” in Brazilian Portuguese) [9].

In order to identify previous experiences and, thus, support the strategic options adopted for the original study of the project, a scoping review was developed, based on the following references: JBI Manual for Evidence Synthesis [10], PRISMA Extension for Scoping Reviews (PRISMA-ScR) [11], Tricco et al. [12] and Munn et al. [13]. The protocol was previously registered on the Open Science Framework platform (https://doi.org/10.17605/OSF.IO/7TBXD, accessed on 18 December 2024).

### 2.2. Inclusion Criteria

Based on the PCC (population, concept, and context) mnemonic [14], the inclusion criteria are as follows:

- “Population”: Observational epidemiological studies (e.g., cross-sectional, case-control, and cohort studies), with no restrictions as to where they were carried out, sampling techniques, or sample profiles. Observing the descriptive nature of the review, it was established that research protocols would also be accepted;
- “Concept”: Use of mixed methods for data collection involving face-to-face approaches and telephone calls;
- “Context”: Studies carried out in the health area, with no restrictions on the theme/subject.

There were no restrictions on the publication date or the use of other data collection methods (e.g., online forms), as long as the study included face-to-face approaches and telephone calls. On the other hand, the eligibility criteria only included studies that were published in English, Portuguese, and Spanish, as well as studies disseminated in peer-reviewed journals.

### 2.3. Identification of Potential Studies

Potential studies were searched for using three strategies: (I) systematic searches were conducted in four databases (PubMed; SciELO; Scopus and Web of Science); (II) searches were conducted in Google Scholar, considering the records of the first 10 pages; and (III) manual searches were conducted in the reference lists of the articles selected for full-text assessment (Supplementary Materials Table S1). More specifically, regarding the work in the databases, the searches were conducted on 26 March 2024, using as a reference the strategy developed for PubMed: (((mixed mode[Text Word]) OR (multi mode[Text Word])) OR (mixing modes[Text Word])) AND (((telephone[Text Word]) OR (phone[Text Word])) OR (call*[Text Word])).

### 2.4. Study Selection Process

The review was conducted by three researchers (AF, LS, and PG), who worked independently throughout the process. PG designed and applied the systematic searches in the electronic databases and Google Scholar. LS and PG were responsible for identifying and removing duplicates, assessing titles, abstracts, and full texts, and extracting data. AF served as the third reviewer and was responsible for assessing the titles, abstracts, and full texts, as well as data extraction, in order to resolve any doubts and establish consensus.

At all stages of the review process, disagreements were resolved through consensus meetings involving the three researchers (AF, LS, and PG). Regarding all disagreements, the researchers presented their justifications, and decisions were made in a dialogical manner.

Data extraction was conducted based on the other experiences of the working group members, using an electronic spreadsheet involving descriptive data (e.g., study name/acronym, objective, subject, country, and year of collection) and methodological data from the included studies (e.g., sample size; gender; age group; sample characteristics; design; study context; implementation; and other strategies used in data collection).

Given the more descriptive focus on MMFT populations, methods, and approaches, from the very beginning of the research, there was no intention to present and/or discuss results. AF, LS, and PG worked together to refine the data in the extraction spreadsheet and draw up the descriptive synthesis. After this stage, the data were exported to the SPSS program, where the descriptive analyses (relative and absolute frequencies) were performed. The refinement and visualization of the descriptive synthesis were conducted by all authors (AF, DU, LS, and PG).

Since scoping reviews present more general objectives (e.g., compared to systematic reviews, which have specific objectives and their implications for practice from a clinical or decision-making point of view), we did not conduct a risk of bias assessment [15].

## 3. Results

### 3.1. Study Stages

**Figure 1:**
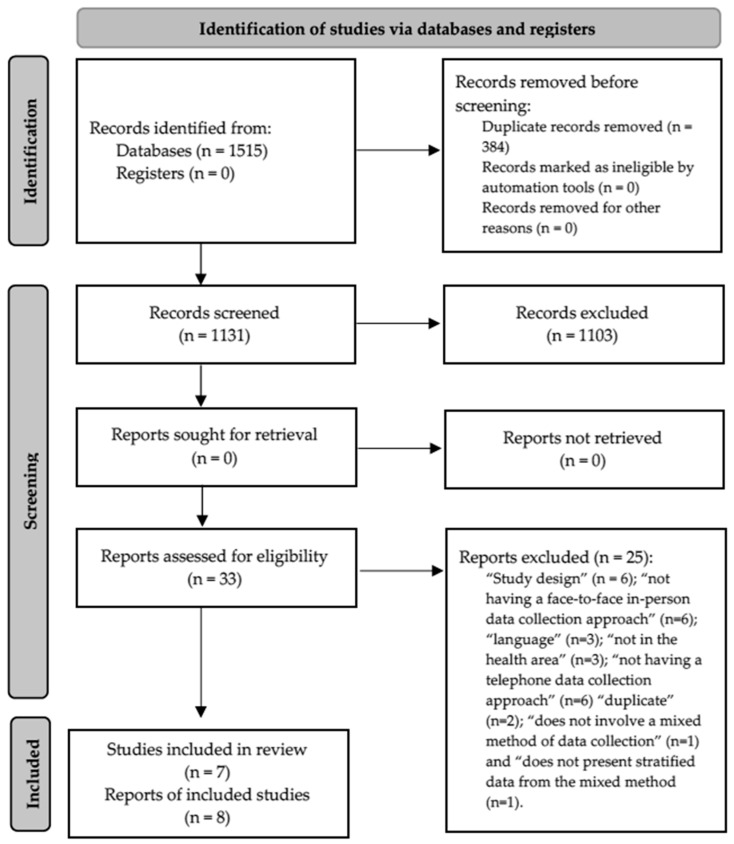
Flowchart of the scoping review.

After removing 384 duplicates, 1131 potential articles were assessed by their titles and abstracts. Of these, 28 remained in the selection process and, with the addition of 5 articles from the complementary searches, 33 were assessed for their full texts. After excluding 25 articles, with the most common reasons being “design” (*n* = 5) and “not having a face-to-face approach” (*n* = 5), 8 articles—containing data from 7 original studies—were included in the descriptive synthesis (Figure 1) [16,17,18,19,20,21,22,23].

### 3.2. Descriptive Information

More specifically, the synthesis contains data from five complete studies [16,17,18,19,20,21] and two protocols [22,23] (Table 1). There was great variation in the topics covered, with the “health of immigrants” theme standing out (*n* = 2) [18,22]. The research was conducted in various countries, and data collection periods were carried out between 1986 and 1992 [17] and 2021 and 2022 [21].

**Table 1:** Descriptive information of the included studies (*n* = 7).

Reference	Study Name	Objective	Topic	Country	Year of Data Collection
Complete studies (*n* = 5)					
Chadiha et al., 2004 [12]	Health Care Financing Administration—HCFA	Presents the methodology, procedures, and results for engaging rural, older African Americans and recruiting their female informal caregivers for a study on well-being and service use.	Healthcare	United States of America	2000–2001
Kelley-Moore, 2006 [13]	Established Populations for Epidemiologic Studies of the Elderly	To systematically compare responses from Black and White older adults in telephone and face-to-face interviews in order to determine whether estimates of racial health inequality vary by survey interview mode.	Health inequities	United States of America	1986–1992
Garrett et al., 2008 [14]	nd	To explore language service provision in a pilot hospital study with two methods of data collection.	Immigrant health	Australia	2004
Rada, 2014; Rada et al., 2024 [15,16]	nd	To present the results of a research study that uses a combination of face-to-face and telephone surveys.	Physical activity and Sports	Spain	nd
Wang et al., 2022 [17]	nd	To explore the current status of palliative care practice for cancer and the influence of COVID-19, from the perspective of oncologists.	Palliative care	China	2021–2022
Study protocols (*n* = 2)					
Koschollek et al., 2023 [18]	German Health Update Fokus—GEDA Fokus	To collect comprehensive data on the health status, social conditions, migration-related factors, and structural factors among people with selected citizenships. This is to enable differentiated explanations of the associations between migration-related aspects and their impact on migrant health.	Immigrant health	Germany	na
Lewis et al., 2023 [19]	People’s Voice Survey—PVS	The PVS is a rapid, population-representative survey that aims to drive action toward more effective and people-centered health systems while promoting health system accountability to the populations.	Health systems assessment	Low- and middle-income countries	na

Legends: na: not applicable; nd: not described.

### 3.3. Methodological Information

Regarding the sample profiles, there was a higher number of studies aimed at the general population (*n* = 6; 85.7%) [12,13,14,15,16,18,19]. However, in terms of ethnic–racial aspects, in addition to focusing on the health of migrants, it is also worth mentioning that two studies involved African Americans [12,13]. By design, there was a predominance of cross-sectional studies (*n* = 6; 85.7%) [12,14,15,16,17,18,19] conducted in community contexts (*n* = 5; 71.4%) [12,13,15,16,18,19] (Table 2).

**Table 2:** Methodological information of the included studies (*n* = 7).

Reference	Sample Size	Gender	Age Range	Sample Characteristics	Study Design	Study Setting	Implementation	Other Strategies
Complete studies (*n* = 5)								
Chadiha et al., 2004 [12]	1547	Both	Older adults	Rural African Americans and their informal caregivers (women)	Cross-sectional	Community	Telephone interview for initial screening (to see if the individual met eligibility criteria) and, subsequently, a face-to-face interview.	none
Kelley-Moore, 2006 [13]	2387	Both	Older adults	Higher frequency of African Americans	Cohort	Community	Seven annual interviews (1986–1992): interviews 1, 4, and 7 were face-to-face residential interviews and the remainder were by telephone.	none
Garrett et al., 2008 [14]	258	Both	Older adults	Most were not proficient in the English language	Cross-sectional	Hospital	Telephone interview (7–14 days after hospital discharge) and review of medical records.	none
Rada, 2014; Rada et al., 2024 [15,16]	600	Both	Adolescents, Adults, and Older adults	Majority of men, aged 35 or under	Cross-sectional	Community	Face-to-face interviews in three districts and by telephone in one district, considering social and geographical characteristics.	none
Wang et al., 2022 [17]	37	Both	Adults	Professionals involved in palliative cancer treatment (mostly men, doctors, and those who work in urban contexts)	Cross-sectional	Hospital	Interviews could be in person, online, or by telephone, depending on the individual’s availability.	Online interview
Study protocols (*n* = 2)								
Koschollek et al., 2023 [18]	33,436	Both	Adults and Older adults	Immigrants residing in Germany (e.g., Croatian, Italian, Polish, Syrian, or Turkish)	Cross-sectional	Community	Face-to-face and/or telephone interviews as alternatives for those who did not respond to the self-completion questionnaire (online or on paper).	Self-completion questionnaire online and on paper
Lewis et al., 2023 [19]	nd	Both	Adults and Older adults	People from low- and middle-income countries, preferably	Cross-sectional	Community	Interviews were preferably conducted by telephone. Implementation could be supplemented by face-to-face interviews or online interviews.	Online interview

Legends: na: not applicable; nd: not described.

Also in Table 2, in relation to the implementation of the MMFT, the strategies indicated different paths, from the use of the telephone as an initial screening, to face-to-face interviews (*n* = 1; 14.3%) [16]; the use of face-to-face interviews and telephone calls, according to the geographical accessibility of the districts (*n* = 1; 14.3%) [19,20]; the choice of one of the techniques according to the availability of the participants (*n* = 1; 14.3%) [21]; and the feasibility of face-to-face or telephone calls in cases where participants did not answer the self-completion questionnaire [22]. In three studies (42.9%), the use of online strategies for data collection (via an interview or self-completion questionnaire) was also observed [21,22,23].

## 4. Discussion

In order to map the information from health surveys that used the MMFT approach in the data collection, the synthesis of this review was composed of data from seven original studies, highlighting cross-sectional designs, involvement of adults and/or elderly people without specific health conditions, and variability between data collection strategies with complementary use of online approaches.

Given the more specific interest in health surveys, from the planning of the review, it was anticipated that the descriptive synthesis would be composed mostly—or even entirely—of cross-sectional studies. It was also expected that there would be a greater number of studies conducted in community settings than in clinical or hospital settings.

As mentioned, this review was designed under the assumption that the MMFT approach has more potential than implementing techniques separately, in different aspects, justifying the non-inclusion of studies that compare different techniques. It is worth noting that the literature suggests differences between the approaches in terms of motivating interviewees to provide accurate answers, reducing the difficulty of tasks [24], and among the elderly, approaches involving memory performance [25].

In addition to these issues, the MMFT approach can also be justified via the increased feasibility of data collection, in the sense of facilitating access or even avoiding losses/dropouts in situations where there is difficulty in dialogue, as in the study by Garrett et al., which mostly involved people with no English proficiency. Perhaps these issues also justify the use of the MMFT approach in studies focused on the health of immigrants [18,22] and the health of the Black population [16,17], considering that these groups are generally situated in greater contexts of vulnerability [26] and inequality in regard to access to health services [27].

However, it is worth noting that the MMFT is not restricted to research conducted among people in vulnerable situations. In the study by Rada et al., for example, telephone collection was carried out to reach people who lived in a higher-income district, characterized by gated and guarded housing, which differentiated it from the characteristics of the other three districts chosen for the research, consisting of blocks of houses and buildings that were more easily accessible, where data collection took place face-to-face.

With regard to the operationalization of data collection, the results showed great variability among studies, with examples including the periodic alternation between approaches [17], selecting the approach according to availability [21], and adopting the telephone approach as an initial screening, followed by the operationalization of the face-to-face approach [16,18].

We believe that this variability in how data are collected is justified by issues of access, considering that most studies deal with vulnerable populations. Given that each option was chosen based on particularities such as identity, geographical or territorial aspects, or the resources and infrastructure available to the research teams, it can be assumed that contextual factors led to this heterogeneity. And these seem to us to be important issues, in order to control possible biases in the self-selection of participants or problems of accessibility in certain groups, and how they can affect the validity of the results.

The additional use of online strategies was one of the points that caught our attention in the studies included [21,22,23]. With the consolidation of digital technologies, the application of online questionnaires has become a possibility for reaching more people, reducing costs [28], and highlighting the existence of large-scale health surveys conducted using online data collection [29,30,31].

However, recognizing that each form of data collection administration has its own particularities, it is important to be cautious about the impossibility of describing the sample and the bias of the answers [32], since respondents can pass the instrument on to people with similar conditions to prepare other answers. When designing data collection based on a probabilistic sample, it is necessary to ensure that the online instruments are sent to and answered by people who have actually been selected for the survey.

Recognizing the state of the art among studies that have used the MMFT approach for data collection allows us to share suggestions for future studies. One of the points to be taken forward involves a more detailed analysis of the success rates between the face-to-face and telephone approaches, since, among the completed studies, little has been discussed about the prerogatives and the extent to which the MMFT approach managed to reach the total number of people initially drawn for the study, in order to control non-response and non-coverage biases. This is a debate that seems important to us, given the indication that the effects of non-response bias are intensified when response rates are lower than 70% [33].

Even so, it is worth recommending the MMFT approach for data collection in future health surveys, not just for more specific populations. The complementarity between the approaches allows for faster, cheaper, and more comprehensive data collection, as long as the implementation of data collection is based on recognizing the contextual conditions that permeate the study (e.g., territories, infrastructures, and access to the target population). In addition, we hypothesize that longitudinal studies can also use the MMFT approach to collect data over the course of follow-up, avoiding losses and dropouts.

However, it is important that future studies indicate the response rate of each type of approach separately, in order to make it easier to understand which strategy had the most potential. For example, in the study by Garrett et al. [18], there was a higher response rate in the multilingual telephone survey. We also suggest that future studies make direct comparisons between MMFT and other mixed-mode data collection approaches.

The main limitation involves the small number of studies in the synthesis, particularly complete studies, which, if there were more of them, would allow for a more in-depth discussion of the aspects mentioned and a greater exploration of the data. On the other hand, the study’s potential lies in its focus on the complexity of the MMFT approach, in order to present the themes, contexts, populations, and how they can be operationalized in health surveys.

## 5. Conclusions

In conclusion, the main characteristics of the studies that used the MMFT approach for data collection include cross-sectional designs, involvement of adults and/or elderly people without specific health conditions, variability between data collection strategies, and complementary use of online approaches. It is recommended that future studies assess aspects of the MMFT approach in terms of response rate, cost reduction, and the speed at which health surveys are carried out.

## Supplementary Materials

The following supporting information can be downloaded at https://www.mdpi.com/article/10.3390/epidemiologia5040054/s1, Table S1: Search strategies used in databases.
