# Supplementary material for: Multi-Mode Face-to-Face and Telephone Approach to Data Collection in Health Surveys: A Scoping Review
Source: Epidemiologia (Basel). 2024 Dec 19;5(4):796–804. doi: 10.3390/epidemiologia5040054 (PMC11675162; doi:10.3390/epidemiologia5040054)
Supplement: Supplementary file 1 [file epidemiologia-05-00054-s001.zip › epidemiologia-3327210-supplementary.pdf]

Supplementary Table S1. Search strategies used in databases

Database	Strategy	Studies
PUBMED	(((mixed mode[Text Word]) OR (multi mode[Text Word])) OR (mixing modes[Text Word])) AND (((telephone[Text Word]) OR (phone[Text Word])) OR (call*[Text Word]))	196
SCIELO	("mixing modes" OR "multi mode" OR "mixed mode")	21
SCOPUS	(TITLE-ABS-KEY ("mixing modes" OR "multi mode" OR "mixed mode") AND TITLE-ABS-KEY (call OR phone OR telephone)) AND (LIMIT-TO (DOCTYPE , "ar"))	351
WEB OF SCIENCE	ALL=("mixing modes") OR ALL=("multi mode") OR ALL=("mixed mode") AND call (All Fields OR phone (All Fields) OR telephone (All Fields) and Article (Document Types)	947
